# Oxidative Stress in Mesenchymal Stem Cell Senescence: Regulation by Coding and Noncoding RNAs

**DOI:** 10.1089/ars.2017.7294

**Published:** 2018-09-20

**Authors:** Rosa Vono, Eva Jover Garcia, Gaia Spinetti, Paolo Madeddu

**Affiliations:** ^1^ Laboratory of Cardiovascular Research, IRCCS MultiMedica, Milan, Italy.; ^2^ School of Clinical Sciences, Bristol Heart Institute, University of Bristol, United Kingdom.

**Keywords:** cell therapy, mesenchymal stem cells, pericytes, reactive oxygen species, senescence

## Abstract

***Significance:*** Mesenchymal stem cells (MSCs), adult stem cells with the potential of differentiation into mesodermal lineages, play an important role in tissue homeostasis and regeneration. In different organs, a subpopulation of MSCs is located near the vasculature and possibly represents the original source of lineage-committed mesenchymal progenitors.

***Recent Advances:*** The plasticity and immune characteristics of MSCs render them a preferential tool for regenerative cell therapy.

***Critical Issues:*** The culture expansion needed before MSC transplantation is associated with cellular senescence. Moreover, accelerated senescence of the total and perivascular MSC pool has been observed in humans and mouse models of premature aging disorders. MSC dysfunction is acknowledged as a culprit for the aging-associated degeneration of mesodermal tissues, but the underlying epigenetic pathways remain elusive. This article reviews current understanding of mechanisms impinging on MSC health, including oxidative stress, Nrf2-antioxidant responsive element activity, sirtuins, noncoding RNAs, and PKCs.

***Future Directions:*** We provide evidence that epigenetic profiling of MSCs is utilitarian to the prediction of therapeutic outcomes. In addition, strategies that target oxidative stress-associated mechanisms represent promising approaches to counteract the detrimental effect of age and senescence in MSCs.—*Antioxid. Redox Signal.* 29, 864–879.

## Introduction

Understanding the aging process and the mechanisms underpinning the development of aging-associated diseases represents one of the most important endeavors of modern medical research. The death rate at all ages has been dramatically reduced in recent decades, resulting in a remarkable increase in life expectancy. However, increased lifespan also correlates with an increasingly old population and an increased number of individuals with chronic pathologies often requiring hospitalization.

Accumulating evidence suggests that stem cells play a crucial role in controlling physiological homeostasis, and rate of aging and stem cell exhaustion is considered one of the hallmarks of aging (67, 69). Hence, maintenance of stem cell health may translate into postponing aging-related diseases. The loss of stem cell activity and acquisition of a senescent phenotype is due to both intrinsic factors, such as DNA damage, telomeres shortening, and chromatin modifications [as reviewed in Ref. (9)], and external factors involving the stem cell microenvironment [cytokine stimulation (74)] and widespread damage in tissues. Senescence has a dualistic function: from one side, it protects cells from damage and oncogene activation but from the other, it limits the possibility for tissue regeneration triggering aging-related deterioration. In the past decades, mesenchymal stem cells (MSCs) have been investigated as therapeutic tools for regenerative medicine, especially for treating chronic diseases. However, the possible impact of pathologic conditions on MSC viability and function has to be taken into account. In addition, MSCs' numerical reduction or dysfunction can *per se* cause pathologies (101). For instance, accelerated attrition of the MSCs pool has been observed in premature aging disorders, including Werner syndrome and Hutchinson-Gilford progeria syndrome (66, 124). It is, thus, clear that these cells, although harboring multilineage regeneration potential, may manifest intrinsic defects compromising their use in regenerative medicine. Conversely, transplantation of young MSCs increases the lifespan and fitness of progeroid phenotype as observed in mice (62). However, the genetic and epigenetic mechanisms underpinning MSC-mediated senescence have not been fully elucidated.

**FIG. 1.**
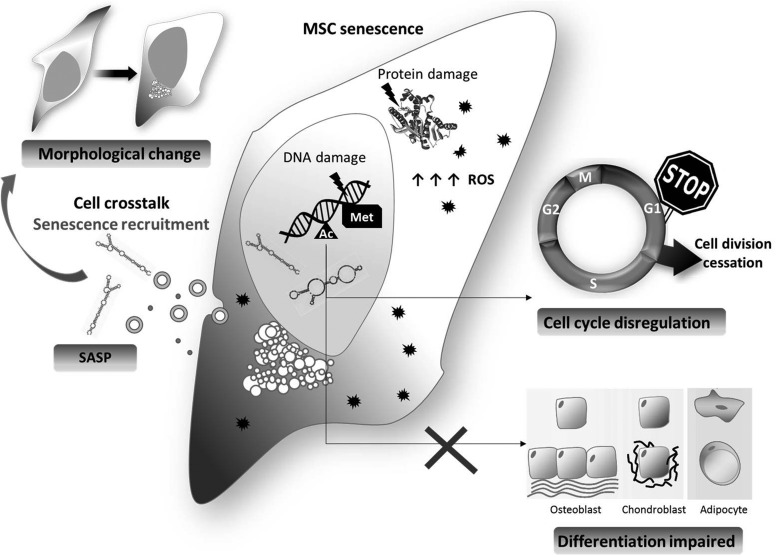
**Overview of senescence in MSCs.** MSC senescence is driven by diverse events, which occur as the cells, proliferates, such as epigenetic modifications, DNA damage, and ROS accumulation. Those events cause an irreversible cell cycle arrest, a change in morphology (spread and enlarged), and an impairment in differentiation ability. In addition, senescent cells produce and secrete a series of SASP paracrinally mediating senescence of close MSCs. MSC, mesenchymal stem cell; ROS, reactive oxygen species; SASP, senescence-associated secretory phenotype.

This article reviews current understanding of the contribution of oxidative stress as a trigger of modifications at DNA and RNA leading to MSC aging and senescence (Fig. 1). We also discuss how a quality and quantity assessment of these aging-related modifications can help upgrade current regenerative medicine approaches.

## The Nature of MSCs and Their Potential for Therapy

In humans, MSCs can be isolated from different adult tissues, including bone marrow where they were first discovered in 1970, skeletal muscle, adipose tissue, umbilical cord, synovium, the circulatory system, dental pulp, amniotic fluid, fetal blood, liver, and lung [as reviewed in Ref. (85)]. Minimal characterization criteria helped to standardize MSC isolation and allowed the definition of MSCs as plastic adherent lineage-negative cells, expressing CD105, CD73, CD90 and potentially able to differentiate at least to osteocytes, chondrocytes, and adipocytes (42). Recently, new criteria, including markers of potency, have been recommended (33). Despite their low abundance [in the bone marrow, their yield spans between 0.01% and 0.001% of nucleated cells (86)], MSCs are believed to be one of the most useful cell sources for clinical application in tissue regeneration. Indeed, compared with embryonic stem cells, MSCs are safe and non-immunogenic.

Although normally in a quiescent state, MSCs can re-enter the cell cycle and differentiate following specific stimuli such as tissue injury. Thus, MSCs are important in guiding the processes of healing and tissue regeneration (83). In addition, MSCs are not only able to give rise to the cell types found in the tissue they were isolated from, but they can also differentiate into a variety of mesodermal cell types and into cell types of other germinal layers through a process known as transdifferentiation (85).

Great relevance has been attributed to perivascular mesenchymal cells, which possibly represents the original source of lineage-committed mesenchymal progenitors. These cells are better known as pericytes. *In vitro*, pericytes show typical mesenchymal properties with the capacity to attach to tissue culture plastic, expand for multiple passages, and differentiate into osteogenic, chondrogenic, or adipogenic lineages (23). *In vivo* lineage tracing studies have reported pericytes as progenitors of white adipocytes (102), follicular dendritic cells (59), and skeletal muscle (25, 26). Pericytes have also been proposed to give rise to neurons, astrocytes, and oligodendrocytes (27) and to play a major role as fibroblast progenitors in fibrotic responses (34, 38). However, in a very recent paper from Guimaraes-Camboa, the mesenchymal origin and properties of pericytes were called into question while using fate mapping experiments in murine models. Using the transcription factor *Tbx18* as an embryonic pericyte marker, Guimaraes-Camboa *et al.* demonstrated that Tbx18-positive cells also co-express pericyte markers CD146 and neural/glial antigen 2, but they do not differentiate during the development in any mesenchymal lineage (40). These results challenge the current view of endogenous pericytes as multipotent tissue-resident progenitors and suggest that the plasticity observed *in vitro* or after transplantation *in vivo* arises from artificial cell manipulations *ex vivo*.

## MSCs in Regenerative Medicine: State-of-the-Art

MSCs represent a great promise for regenerative medicine. They can help in the repair of injured organs and their functional recovery by migrating and homing at the injury site (71, 109). This can be achieved by both transplanting purified MSCs from a donor (especially in a syngeneic fashion) and stimulating their activation *in vivo* from the reservoir located *in situ.* Moreover, MSCs can repair injured tissues by directly differentiating to functional cells of the tissue, favoring angiogenesis or paracrinally, stimulating resident progenitor activation (90). Paracrine factors released from transplanted or resident MSCs can also contribute to immunosuppression of the host to avoid an immune response. This effect seems to be exerted by both paracrine suppression of lymphocyte T and favoring the transition of macrophages from subtype M1 (pro-inflammatory) to M2 (anti-inflammatory). Moreover, MSCs secretome is also involved in paracrine protection against apoptosis and oxidative stress [reviewed in Ref. (64)]. In recent years, the role of microparticles as a component of MSCs secretome has been envisioned as a new tool in regenerative medicine. Those particles promote the horizontal transfer of mRNAs, microRNAs (miRs), and proteins modulating the activity of target cells (84). For instance, studies defined the important role of exosomes in mediating paracrine information transfer to healing myocardial ischemia/reperfusion injury in mice (61) and pigs (105). Despite the bulk of information available on the feasibility and therapeutic outcomes, MSC-based cell therapy did not obtain the expected success in clinical practice. This is due to several factors such as documented recovery of immunogenicity associated with MSC differentiation causing late rejection (45), the poor engraftment (58), the short-term therapeutic benefits (95), and the acquisition of a senescent phenotype after several cycles of expansion *in vitro.* Senescence, in particular, is due to the interplay between stochastic and programmed events (41). MSC senescence is triggered by a plethora of events that are mostly regulated by epigenetic phenomena, which will be herein discussed.

## MSCs are not Exempted by Experiencing Aging and Senescence: Regulating Mechanisms

### Cell *vs.* organism senescence

Despite their self-renewal ability, MSC expansion is restricted by the Hayflick limit, that is, the number of times a cell can divide until senescence occurs. At this point, cell cycle irreversibly stops even though MSCs are still metabolically active. Morphologically, senescent MSCs lose their typical spindle-like shape and become flat and enlarged with the formation of senescence-associated heterochromatic foci linked to the repression of proliferative genes and, hence, DNA synthesis. The mentioned morphological changes reflect a functional impairment since cells lose their differentiation potential, thus limiting their therapeutic capacities (114). In a sense, senescence is the recapitulation, in the cell, of aging in individuals. However, controversy still exists regarding the direct contribution of senescent MSCs to organism aging. In any case, senescence is a physiologic event and it is the expression of the lack of perfection in biologic systems. Indeed, every time a cell divides DNA damage accumulates at specific chromosomal regions known as telomeres. Telomeres are complexes made of proteins and single-stranded nucleotides located at the end of every chromosome and are more sensitive to replication damage stress than other chromosomal regions (96, 99). Their instability is due to the inability of DNA-polymerase to work on single-stranded sequences. The consequence is a shortening of telomeres at every cell division till reaching a critical threshold that triggers senescence. Telomeres attrition is generally counteracted by telomerase reverse transcriptase (TERT), an enzyme that is able to add DNA sequences at telomeric ends of chromosomes by using an RNA template. However, telomerase is often poorly expressed in human MSCs and in any case, it cannot counteract other causes of senescence (9) herein described.

### Epigenetic regulation

Changes in the epigenetic regulation of gene expression have been recently related to the senescent phenotype. Koch *et al.* found that DNA methylation varied by 40% between early and late passages, with a general tendency to hypomethylation but, in some cases, also hypermethylation was detected. The same group proposed an Epigenetic-Senescence-Signature (ESS) based on the methylated state of specific CpG islets. In particular, they found that two CpG were hypermethylated whereas four were hypomethylated. This ESS significantly correlated with cell passage and was found with the same characteristics in MSCs from different origins (58). The ESS could, thus, be a helpful tool to evaluate when MSCs go toward senescence and standardize the good manufacturing practices (GMP) procedures of *in vitro* expansion before cell therapy as further discussed later in this article.

### Senescence associated secretory phenotype 

Other causes of MSC senescence are sustained cytokine stimulation and secreted autocrine or paracrine factors, including cytokines, growth factors, proteases, and soluble receptors called senescence-associated secretory phenotype (SASP). It has been demonstrated that chronic stimulation with antiproliferative cytokines such as interferon-β and transforming growth factor-β in MSCs induces ROS-mediated p53 or p16^ink4a-^dependent senescence (74, 118). More recently, Jin *et al.* showed that senescent human umbilical cord blood-derived MSCs secrete SASP molecules, among which the most dominant was monocyte chemoattractant potein-1 (MCP-1/CCL2) that was able to induce senescence through binding to its cognate receptor CCR2. In this case, the MCP-1 release is epigenetically regulated by a decreased level of BMI1, a member of the polycomb repressor complex-1 (49). Hence, MCP-1 could be exploited as a marker of cell senescence both *in vitro* and *in vivo*. Since MCP-1 is secreted also by other mural cells such as vascular smooth muscle cells (VSMCs), an MSC-specific secretome signature needs yet to be identified. Of note, we reported an increase of MCP-1 expression in aortic VSMCs from old rats and humans, pointing at a central role of this chemokine in age- and senescence-associated cellular dysfunctions (100). Lastly, SASP likely contributes to the correlation between senescent cell accumulation and disease onset (125).

### Other mechanisms 

**FIG. 2.**
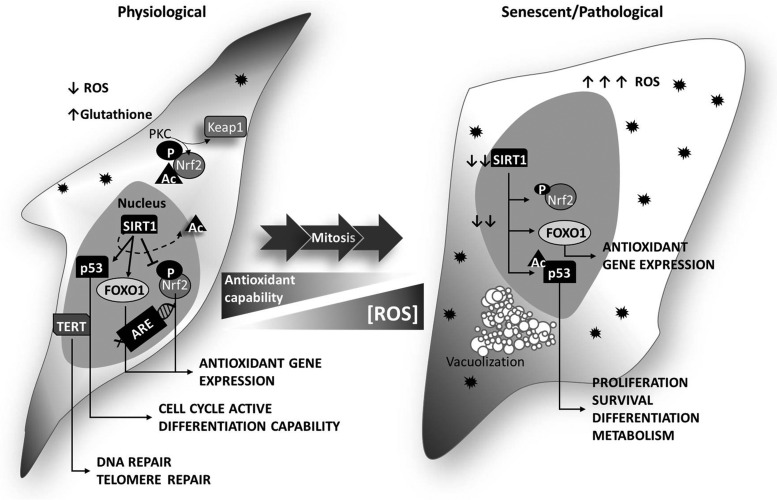
**MSC senescence: correlation between ROS and senescence regulators.** MSCs (*left*) show a physiologically low ROS content (*black spiky dots* in the figure), which is important to regulate cell proliferation and differentiation. After a series of cell divisions, ROS increase, due to an impairment of the radical defense system, and accumulate into the cell (*right*). ROS increase induces the overexpression of p53, which, once in the nucleus, is easily acetylated (^Ac^p53). Although in functional MSCs nuclear p53 can be deacetylated by SIRT1, in senescent cells SIRT1 is downregulated, thus maintaining p53 acetylated and inactive. ARE, antioxidant responsive elements; SIRT1, sirtuin 1; TERT, telomerase reverse transcriptase.

Whatever the trigger is, the effectors of MSCs senescence are molecules governing cell cycle progressions such as p53 and p16^ink4a^-pRb. The two pathways act in both a concerted way and independently and different are the stimuli-activating one pathway, the other, or both (9, 65) (Fig. 2). *In vitro*, senescence is accelerated by the oxygen tension used for culturing conditions: MSCs are generally cultured at 21% O_2_, a percentage that is 4 to 10 times higher than the tension they experience inside the tissue of origin. The excess of oxygen increases the production of reactive oxygen species (ROS), which, in turn, activate senescence-associated pathways (30).

## The Oxidative Stress Theory

ROS are physiological byproducts of the oxidative metabolism produced during the passage of the reduction equivalents through the mitochondrial electron transport chain. Low physiological ROS concentration is beneficial to promote proliferation, DNA stability, and cell survival. This is particularly true for MSCs: at a steady state, MSCs rely on glycolysis and have a low content of ROS as reviewed in Ref. (92). In addition, they also have a high content of glutathione, and an active antioxidant machinery (111). However, as they replicate, intracellular and extracellular senescence-triggering stimuli induce a marked production of ROS. High ROS levels damage proteins and DNA, especially at a telomeric level inducing the so-called replicative senescence (48) that are otherwise known as stress-induced premature senescence (108). In this ROS-centric vision of MSC senescence reported as *oxidative stress theory,* ROS act as a molecular “grenade” affecting all cellular macromolecules at the multi-compartmental level, triggering senescence-associated pathways (15).

### ROS-associated pathways 

The increase in ROS content also correlates with a decrease in cell differentiation except for adipogenic differentiation that results in an increase. In addition, under high ROS content, the depotentiation of scavenger systems was observed. This phenomenon is due to a decline in the activity of the transcription factor *Nrf2*, which regulates ROS scavenger expression (70). Under physiological conditions, Nrf2 is sequestered in the cytoplasm bound to Keap1 protein. Increased ROS concentration induces Keap1 detachment and Nrf2 phosphorylation. Huang *et al.* demonstrated that protein kinase C (PKC) directly leads to Nrf2 activation by phosphorylation (44). Phosphorylated Nrf2 translocates to the nucleus where it promotes transcriptional activation of antioxidants scavenging enzymes (heme oxygenase-1 [HO-1], NAD(P)H:quinoneoxidoreductase 1 [NQO1], catalase, and superoxide dismutase [SOD]) binding to the antioxidant responsive elements (ARE) in their promoter regions (19). However, the Nrf2 expression is drastically reduced in senescent cells and aging in general (5), thus contributing to increased redox imbalance. Recently, our group found that a specific PKC isoform, PKCβII, plays a central role in diabetic complications. PKC is one of the members of a wide family of serine/threonine kinases. After activation by growth factors and ROS, PKC becomes active, thus regulating diverse pathways through a phosphorylation cascade. In our work, PKCβII amplifies oxidative stress in muscular pericytes (MPs) that are isolated from diabetic complicated skeletal muscle. Our results suggest that diabetic MPs share common traits with senescent cells since they showed: (1) a slowed replicative capacity, (2) a reduced myogenic differentiation potential counteracted by an increased propensity to adipogenesis, (3) a repression of antioxidant systems, and (4) increased ROS burden. Such a functional impairment is attributable to ROS-guided activation of the PKCβII-p66^Shc^ signaling pathway (113). In this sense, diabetes not only recapitulates but also exacerbates cell senescence.

## New Players in the Control of MSC Healthy Status: Noncoding RNAs

New post-transcriptional regulators associated with senescence include RNA binding proteins (RBPs) and noncoding RNAs of both miR and long noncoding RNA (lncRNA) classes. Indeed, it is becoming increasingly clear that in addition to coding genes, also noncoding RNAs regulate gene expression. MiRs are small, about 20 nucleotides in length, double-strand RNA found intracellularly and extracellularly, which interfere with RNA translation *via* different mechanisms, with the better known implying the binding to the 3’UTR sequence of the target gene RNA and the inhibition of translation *via* destabilization/degradation of the transcript. In the nucleus miR precursors, pri-miRs are digested by polymerase II and III to give rise to pre-miRs that are sequentially cut by the enzyme Drosha and transported to the cytoplasm where mature miRs are generated by the action of Dicer and enter the machinery of mRNA control. Aging and oxidative stress have been associated with dysregulation of the fine-tuned miR biogenesis process. Dicer, for example, is reportedly downregulated in old rat endothelial cells (ECs) compared with young donor cells (110). The consequent decrease in miRs content impacts cell capabilities to proliferating, adhering, migrating, and networking as shown in an experiment in which Dicer was reciprocally inhibited or overexpressed in young and old ECs, respectively. In mice, aging affects the composition of miRs in the adipose tissue, a central player in the control of lifespan and diseases. Of the total detected miRs by Mori *et al.*, 51% decreased and only 10% of miRs increased when comparing 3-, 6-, and 24-month-old animals. The authors indicate a global decline of the miR-generating machinery, including Exportin-5 (the enzyme that transports pri-miRs from the nucleus to the cytoplasm) and Argonaute-2 (a miR-binding protein), but the most affected is Dicer. The same authors evidenced that Dicer Knockout cells show signs of senescence such as β-galactosidase positivity and upregulation of genes controlling mitochondrial stress pathways (75). Dicer down-modulation was confirmed in preadipocytes from old human donors and in the old nematodes model, pointing at an evolutionarily conserved mechanism. Dicer impairment was efficiently reverted by calorie restriction, a strategy that proved beneficial in extending lifespan cross-species (31). Of note, in isolated preadipocytes, oxidative stress induced by H_2_O_2_ treatment resulted in Dicer decrease, an effect prevented by insulin treatment.

Also, Exportin-5 and Drosha can be influenced by oxidative stress, although the associated mechanisms are still not completely clear (22, 103).

On the other hand, lncRNAs are RNAs of >200 nucleotides in length that do not code for proteins (72) comprehending antisense, intronic, and intergenic transcripts but also pseudogenes and retrotransposons. The question as to whether lncRNAs are just byproducts or are actively generated to control cell biology is still open. Growing evidence shows that they can influence gene expression in multiple complex manners, interacting with DNA, RNA, or RBPs. LncRNA effect is at the transcriptional and post-transcriptional level, and they can influence translation while acting as “sponges” for miRs and proteins (13, 63).

### miRNAs: cellular effects

**FIG. 3.**
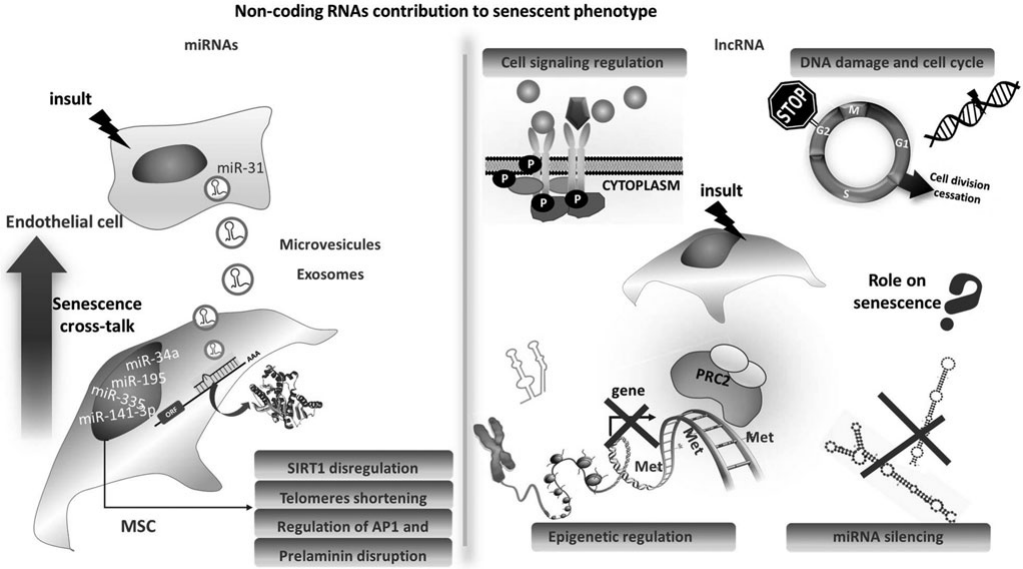
**Noncoding RNAs' contribution to senescent phenotype.** Several micro RNAs have been demonstrated to regulate senescence-related mechanisms (*left*). One of them, miR-31 has been reported to be shed from senescent endothelial cells as microparticle cargo. Once miR-31 is taken up by MSCs, it is sufficient to induce cellular senescence. Less clear is the contribution of lncRNAs to senescence for which many mechanisms of action are possible, as depicted here (*right*). lncRNA, long noncoding RNA; miR, microRNA.

The role of noncoding RNAs in cellular senescence has been elegantly reviewed recently by Abdelmohsen and Gorospe (1); therefore, we will focus here only on some MSC-specific aspects (Fig. 3). Overall, miRs are known to control senescence-associated pathways such as the p16/Rb, the p53/p21, and secretory moieties related to the aging phenotype. A specific pattern of differentially expressed miRs has been associated with senescence in cells, including MSCs (35, 60). As mentioned earlier, one of the issues about noncoding RNA is still related to dissecting whether changes in their profile play an active role in the onset of pathologies or they just mirror the senescence of cell and tissue becoming dysfunctional. Some miRs have been validated for their crucial function in MSCs. miR-195 is one of them. It has been found to be elevated in association with MSCs aging and senescence together with miR-140, miR-146a/b, but miR-195 functional role analysis highlighted a direct control in telomere length as further discussed in this article (78). A functional role of the miR-141-3p increase in senescent MSCs was also demonstrated by linking it to prelamininA accumulation *via* miR-141-3p target ZMPSTE24 (122).

miR-335 and miR-452 are two miRs that are upregulated in old MSCs from adipose tissue (81). MiR-335 has a central role in controlling MSCs proliferation and migration as further evidenced by the group of Tome *et al.* (106, 107). The authors link the cellular effect of miR-335 to AP-1 activity and to the regulation of members of the Fos family. Interestingly, the secretome of miR-335-MSCs was globally augmented, pointing at a transition toward the classical SASP condition.

The forced expression of miR-335 in MSCs *in vitro* recapitulates several aspects of senescence, including the impairment in the redox control system, such as the reduction in expression of SOD2 and an increase in superoxide generation. In this respect, it is crucial to note that miRs can themselves control the generation of ROS by regulating the Nrf2 pathway as reviewed in Ref. (21). On the other hand, in response to oxidative stress, a specific pattern of miRs is generated, with the prototype being miR-210 (14, 36). After an increase in the level of hypoxia-inducible factor-1 protein and of miR-210, the two factors regulate each other in a feedback loop. This virtuous cycle is needed for MSC survival in high ROS culture conditions (17, 54).

In a recent article, miR-210 modulation was linked to the beneficial effect in protecting MSCs from apoptosis of a redox controlling small molecule called zeaxanthin dipalmitate (68).

MiR-34a has been largely associated with aging and cardiovascular diseases in particularly affecting the function of cardiovascular cells (7). Confirmation that an miR-34a-dependent modulation of the SIRT-1/FOXO3a pathway is controlling MSCs vitality similarly to other cell types, including ECs, has been recently demonstrated (46, 119, 123).

### miRNAs: paracrine functions 

**FIG. 4.**
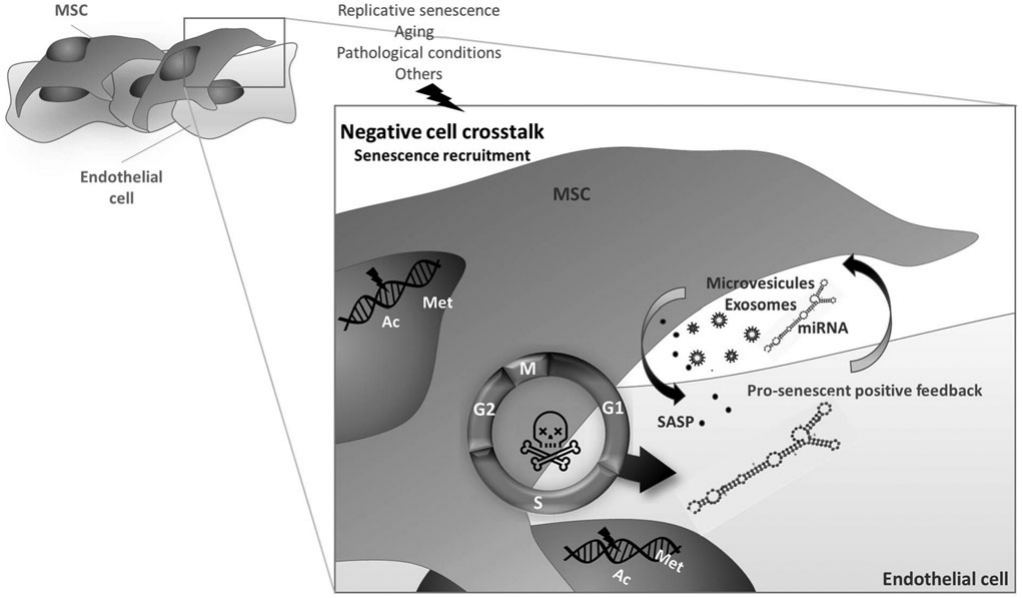
**Negative influences**. Endothelial cells are very sensitive to senescence and secrete molecules and microparticles that are paracrine hallmarks of senescence. Those include SASP, miRs, exosomes, and microvescicles that induce senescence of MSCs. miRs are also shed as microparticle cargo. Senescent MSCs, in turn, influence endothelial cells with similar mechanisms; however, the involvement of miRNA is not that clear.

MiRs exert their action in the cell of origin but also in a paracrine fashion after secretion and transfer to a target cells/tissue. This concept has proved true also in the context of senescence where secreted miRs can vehicle their action from ECs to MSCs/pericytes *via* microparticle shuttling (117). The two cell types are in thigh connection and are reciprocally influenced as we also recently reviewed. ECs are strongly affected by age and senescence, and an SASP condition could apply to ECs too (11, 28) (Fig. 4). One such example of extracellular shuttled miR is miR-31, a senescence-increased miR known to regulate osteogenic differentiation (4) that has been shown to be taken up by MSCs after being released in EC-derived microparticles. The inhibition of miR-31 target gene Frizzled-3 is associated with osteogenic impairment. This effect was paired with an age-associated increase in circulating miR-31 in humans. A similar effect was described for miR-503, a miR that we found to be dysregulated in diabetes, a pathological condition associated with aging and senescence of ECs, which is transferred from ECs to pericytes inducing dysfunction (10, 12).

### LncRNAs

Several hundred lncRNAs are currently known, but their function is still not completely understood. A screening in fibroblast demonstrated that the short form of lncRNAs is upregulated in senescent cells compared with the long-sized ones. This result reinforces the hypothesis that mRNA stability is altered with aging (2, 29). Among the emerged lncRNAs associated with senescence, one that has been investigated in MSCs with functional consequences is HOTAIR, an lncRNA associated with a chromatin locus that is important for epigenetic changes and that is altered in cardiac disease of aging (37, 89). In the article by Kalwa *et al.*, the expression level of HOTAIR is not changed with *in vitro* senescence, but its modulation alters the adipogenic differentiation ability of MSCs. The observed impact on the DNA methylation profile of HOTAIR was accompanied by triple helix formation (RNA-DNA-DNA) in the downregulated genes after HOTAIR overexpression (51).

We expect that several known and new lncRNAs will come into the list of senescence controlling factors in the next future since this class of molecules is the object of intense research.

## Epigenetic Screening Integration with Clinical Data Has a Potential Predictive Value for Identification of Responders to Cell Therapy

**FIG. 5.**
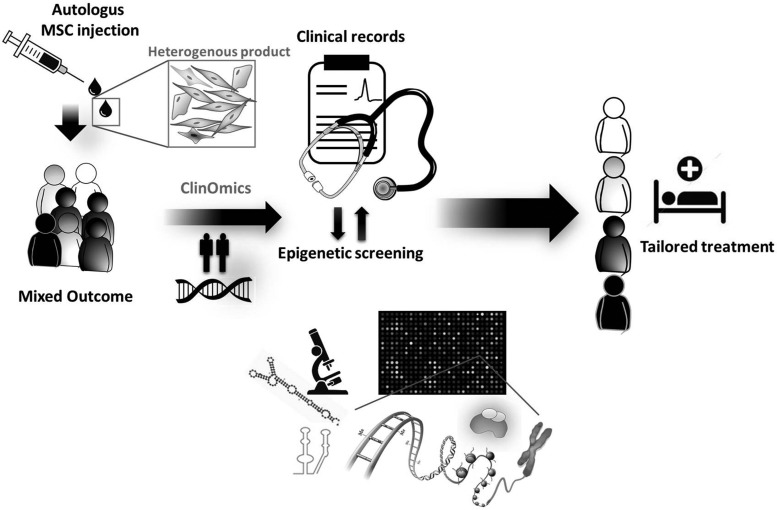
**Deconvolution of patient heterogeneity.** Individual variability in drug efficacy and safety is a major challenge in current clinical practice. In the case of autologous cell therapy, the medicinal product is not homogeneous, and its therapeutic activity may vary among cell lines. Integration of clinical data with epigenetic screening can help allocate patients to the right treatment.

Since MSCs are poorly represented in human tissues, culture expansion is always necessary before the use of MSCs for cell therapy. This raises the question as to whether the occurrence of senescence can be monitored to avoid administration of senescent, non-mitotic cells, which would drive cell therapy failure. Patient-derived MSCs display heterogeneous reparative capacity, leading to unpredictable therapeutic outcomes and reducing a rigorous clinical application in the whole patient population. As mentioned earlier, attention is now focused on establishing quality control assessment at different levels of the standard operating procedure for harvesting, isolation, and expansion of cell populations, and on identifying predictors that are able to distinguish responders *versus* non-responders, thereby informing personalized therapies (Fig. 5). Hemodynamic predictors failed to pass the initial validation stage. For instance, the importance of basal contractility indexes such as left ventricular ejection fraction (LVEF) in influencing the outcome of cell therapy remains controversial. Two recent meta-analyses of bone marrow cell therapy trials in patients with acute MI indicate that patients experienced similar improvement in LVEF regardless of the baseline LVEF. However, improvements in LVESV were more pronounced in patients with lower baseline LVEF (3, 8). In contrast, in trials of chronic myocardial ischemia, the increase in LVEF elicited by cell therapy was significant only in the group with lower LVEF at baseline (8). Remarkable advancement in omics technology integrated by the interface with clinical data, for example, the clinomics, promises to bridge basic biological data and benefits on human health. Clinomics-based interrogation of stem cell heterogeneity may help deconvolute the heterogeneity of reparative performance, thus informing the development of new high-fidelity protocols (104).

Biological markers of MSCs have been already validated as predictors of response to autologous stem cell transplantation in patients with neuronal degenerative diseases (55). There is still a paucity of data regarding the use of epigenetic screening to assess treatment response of cardiovascular MSC therapy. We found that a patient's age and smoking habit are negatively associated with therapeutic outcomes of pericyte cell therapy in a mouse model of limb ischemia, suggesting that intrinsic and environmental determinants of senescence could impact cell therapy performance (39).

### Epigenetic screening

To identify new epigenetic predictors of therapeutic activity, we performed a whole genome DNA methylation array of the human pericyte populations (39). Methylation regulates gene expression by different mechanisms, acting at the promoter region and gene bodies. We identified 936 unique genes (106 of these involving promoter regions), whose methylation status is correlated with the blood flow recovery from ischemia. In addition, 5461 genes (930 in the promoter region) had a methylation status that correlates with capillary density in the ischemic muscle, and 784 unique genes (of these 89 in the promoter region) were associated with arteriole density. Integration of all the differentially methylated genes associated with the three outcomes identified 304 genes, of which 158 (52%) bear KROX-/EGR1-binding sites. The transcription factor KROX/EGR1 couples short-term changes in the extracellular environment to long-term changes in gene expression. It is induced by different growth factors and chemokines, including VEGF and stromal cell-derived factor (SDF-1), and stimulates microvascular neovascularization through FGF-mediated mechanisms. Moreover, the Arf-EGR-C/EBPβ axis is an important determinant of cellular responses (senescence or transformation) to oncogenic Ras signaling (91). An analysis of the genomic locations of the 304 genes shared by the three therapeutic outcomes identified a significant enrichment of the 6p21 loci for a gene network centered on CREB-binding protein. Such a nuclear protein binds to CREB, which restricts cellular senescence and apoptosis (94) and comprises Runt-related transcription factor 1, which belongs to a gene family that is implicated in stem cell plasticity (115).

The mechanisms that concentrate methylation to specific sequences and loci in the genome are unknown, although an interaction between DNA methyltransferases and other epigenetic factors has been proposed (87). Interestingly, a similar clustering has been observed in studies of gene polymorphisms. More than 90% of the genome lacks any disease-associate loci according to a meta-analysis of Genome-Wide Association studies of age-associated diseases (47). Surprisingly, a large spectrum of diseases maps to two specific loci 6p21 and *INK4/ARF* tumor suppressor locus. The former is where the major histocompatibility (MHC) locus resides. Genes at this locus determine a high susceptibility to a variety of auto-immune diseases and diabetes (47). It is not known whether mutations at the MHC locus can alter the immune privileged profile of MSCs, which usually express low levels of MHC class I (MHCI). In addition, 6p21 emerged as a new locus associated with coronary artery disease (CAD) at a genome-wide significance from a comprehensive analysis of the extent of pleiotropy of all CAD loci (116). Altogether, these reports indicate that polymorphic variants and epigenetic modifications at 6p21 loci may have a strong impact on age-associated diseases and regenerative processes.

In our study on human pericytes, the majority of differentially methylated CpG sites were associated with a known transcript. Hence, we next investigated the expressional profile by using gene arrays (GEO accession number: GSE57964) and reverse transcriptase-polymerase chain reaction (RT-PCR) analyses. This was followed by a gene set enrichment analysis to identify transcription factors whose targets are significantly enriched among genes correlating with cell therapy outcomes in the limb ischemia model (39). MAZ, a transcription factor that emerged from the DNA methylation analysis described earlier, was associated with a high number (139) of differentially expressed genes. MAZ is a zinc finger transcription factor that binds to GpC-rich cis-elements in the promoter regions of numerous mammalian genes and is also able to act as a recruiting scaffold for different proteins, such as methylases and acetylases, to the transcriptional complex, thereby acting as an initiator or terminator of transcription (98). The transcription factor plays a role in VEGF-induced angiogenesis under the control of microRNA-125b, of which MAZ is an inhibitory target (88, 97). Intriguingly, the expression of microRNA-125b in pericytes is inversely correlated with their ability to induce reparative vascularization in the mouse limb ischemia model. The consensus sequence of MAZ-binding sites is very similar to that of Sp1-binding sites. In fact, MAZ and Sp1, an anti-senescence transcription factor, bind to the same cis-elements in the promoters of the genes for endothelial nitric-oxide synthase (eNOS), and the receptor for parathyroid hormone. However, MAZ acts as a repressor and Sp1 as an enhancer of eNOS levels, suggesting that they have dual functions in the regulation of gene expression. This contrasting action of different zinc-finger proteins binding to the same cis-elements is attributable to their capacity to recruit different proteins, such as methylases and acetylases, to the transcriptional complex (98). However, screening MSCs' epigenetic modification alone could be not sufficient to yield a homogeneous population. Attention should be also devoted to the microenvironment in which MSCs are grown. ROS load is strictly related to the composition of the culture medium and ROS amount holds the balance of power between cell differentiation or stemness, proliferation, or cell cycle. As the main nutrients involved in ROS production are glucose and oxygen, their concentration and combination should be precisely defined and maintained constant, especially during GMP procedures (32, 56).

## The Long Road from Trigger Signals to Senescence Effectors Passes Through the Control of ROS

In senescent MSCs, ROS increase is paralleled by a decrease of Sirtuin 1 (SIRT1) mediated by its post-transcriptional modulation (93). Studies reveal that sirtuins (SIRT1-7), a family of histone deacetylase, act as life-span-regulating proteins and are master regulators of telomeres maintenance [SIRT1 (18) and SIRT6 (120)] and DNA repair after oxidative stress [SIRT6 (80)]. SIRT1, a nuclear isoform of SIRT proteins, modulates the activity of different proteins involved in the cell cycle. p53 is one of the main targets (121). In particular, SIRT1-dependent p53 deacetylation induces p53 inactivity and thus favors cell cycle progression (112). However, high ROS levels mediate, on the one hand, SIRT1 downregulation, and on the other hand, the upregulation of p53 protein. In this scenario, p53 is also kept in its acetylated and inactive state due to the lack of SIRT1. In such a condition, the cells experience the exit from the cell cycle and, hence, senescence (79).

Another target of SIRT1 activity is FOXO1, a transcription factor involved in the expression of antioxidants enzymes such as SODs and catalase. FOXO1 activity is regulated by the post-transcriptional mechanism such as acetylation. During senescence, the low SIRT1 levels impede FOXO1 deacetylation, which is necessary for its translocation to the nucleus. As a consequence, the antioxidant machinery under the senescence condition is repressed (57). Studies report a direct interaction between SIRT1 and Nrf2, the activity of which ends up with the upregulation of multiple antioxidant enzymes as previously reported. Analysis of the transcriptional complexes binding to ARE sequence detected histone acetyltransferase p300/CBP along with Nrf2 possibly mediating its acetylation. Acetylation of Nrf2 is important to enhance Nrf2 binding to ARE sequences. SIRT1, on the contrary, decreases acetylation of Nrf2 as well as Nrf2-dependent gene transcription (53) as a compensatory mechanism, even though conflicting results exist. Obviously, senescent mesenchymal cells lose such a regulatory mechanisms due to the reduced expression of both proteins. So different effectors, such as FOXO1 and Nrf2 contribute to the increase and maintenance of ROS during senescence, and this process is mediated by SIRT1. As discussed in the next paragraph, resveratrol has been proved to activate SIRT1 overexpression in senescent cells (50). It seems that acting at the SIRT1 level could be a strategy to potentiate SIRT1 and counteract senescence.

## Strategies to Counteract Senescence and Improve MSC Health and Therapeutic Potentials

**FIG. 6.**
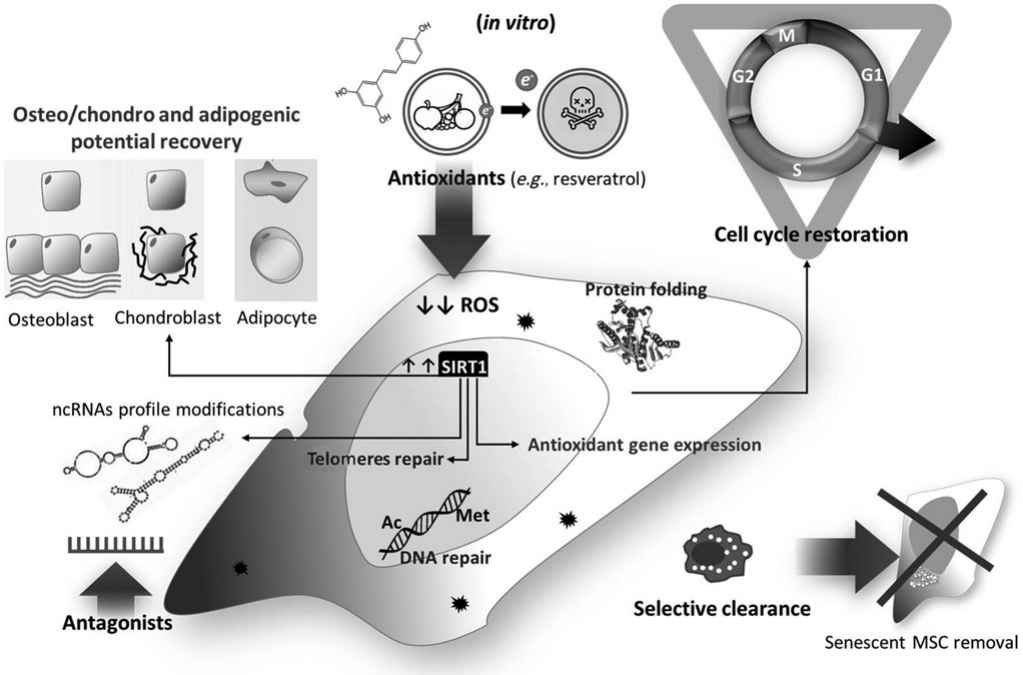
**Strategies to reverse senescence**. MSCs' senescence can be counteracted efficiently by using different approaches: potentiating antioxidant defense, inhibiting senescence-associated RNAs through specific antagonists, and increasing clearance of senescent cells by phagocytes. These intervention strategies induce the decrease of ROS and DNA damage, helping the cell to overcome the cell cycle blockade, improve MSC differentiation potential, and favor morphological recovery.

Taking into account all the mechanisms just mentioned, it appears evident that ROS are the main characters of a play (senescence) with a very intricate plot, regulated thanks to the interaction among sirtuins, p53, FOXO1, and Nrf2. Strategies to treat or reverse senescence of MSCs could help to counteract aging and cell senescence-associated diseases (Fig. 6). The efforts should be directed to both isolated cells for regenerative medicine and *in vivo*. A schematic summary of the mechanisms associated with senescence is presented in Table 1.

**Table 1.** Documented Causes of Mesenchymal Stem Cell Senescence

in vitro	*Reference*	in vivo	*Reference*
High O_2_ tension in culture	29	SASP	87, 61
DNA methylation variation	55	PROGERIA	59, 63, 121
Cytokines, growth factors	71, 115	Exosomes	58, 102
SASP	47, 96–122		
Noncoding RNA	20, 14–34		
Exosomes	114		
ROS	46, 105		

ROS, reactive oxygen species; SASP, senescence-associated secretory phenotype.

### Resveratrol 

Recent studies indicate that resveratrol (3,5,4′-trihydroxy-trans-stilbene), a natural phenol produced by several plants in response to injury, does activate SIRT1 by allosteric interactions that increase SIRT1 affinity for both NAD+ and the acetylated substrate (43). In addition, resveratrol treatment improves the osteogenic and adipogenic differentiation potential of MSCs (24). In this sense, resveratrol mimics the effects of calorie restriction and, thus, could contribute to extending lifespan. Reversing SIRT1 activity could ameliorate different aspects linked to senescence, such as increased antioxidant enzyme expression through interaction with FOXO1 and reactivation of telomerase activity to counteracting telomeres attrition. Moreover, resveratrol treatment has been associated with changes in miRs' profile, supporting the central role of miRs tuning in controlling cellular fate (76, 77).

However, studies in animal models showed that resveratrol supplemented at a later life stage was ineffective (73). Moreover, long-term exposure to resveratrol increased MSCs' senescence. It is becoming clear that resveratrol effects can be beneficial or detrimental depending on the dosage and duration of the treatment. In addition, bioavailability after oral administration in mice and humans should be taken into account to evaluate the good or bad role of resveratrol treatments. Indeed, deeper analysis of blood resveratrol concentration, after oral administration both in mice and in humans, revealed that it is usually lower than the dosage proved to be effective *in vitro*, thus adding a bias in the interpretation of results (82).

### Noncoding RNAs

Another approach to counteract senescent is modulation of miRs known to target molecules that directly or indirectly regulate senescence. Okada *et al.* recently identified in the bone marrow of old mice a population of mesenchymal cells with features of young mesenchymal cells (YMSCs). Microarray analysis identified a different pattern of miR in YMSCs with respect to their old counterpart (OMSCs). Relevant was the identification of miR-195 that was significantly overexpressed in OMSCs. Interestingly, a target gene of mir-195 is TERT. Thus, downregulating miR-195 rejuvenated OMSCs by counteracting telomeres attrition (78). MiR modulation can be employed to boost the therapeutic capabilities of MSCs as we showed studying human saphenous vein-isolated pericytes in a recent work. Transplantation of pericytes in mice infarcted heart resulted in improved cardiac function, blood flow recovery, and angiogenesis *via* paracrine signals activation dependent on miR-132 (52).

More controversial is the role of long noncoding RNA in mediating reversion of senescence. The lack of knowledge about their specific function and the multiple ways of interaction with nucleic acids and proteins render it difficult to target them.

### Senescence clearance 

Clearance of senescent stem cells (SCs) has been exploited in a progeroid mouse model using a transgenic approach, and it reportedly delays several age-associated disorders (6). Further, selective clearance of senescent SCs by a pharmacological agent (ABT263) resulted in the rejuvenation of the stem cell pool in bone marrow and skeletal muscles (16). In this context, new strategies to foster the clearance of damaged stem cells are emerging. Recently, Cheng *et al.* demonstrated that prolonged fasting followed by re-feeding exerts a pro-regenerative effect on bone marrow hematopoietic stem cells in mice and humans (20). Whether this strategy could be useful for mesenchymal stem cells remains unexplored.

## Conclusions

The response of cells to time (organism age or passages in culture) by slowing proliferation/differentiation and acquiring a secretory phenotype represents the effort to counteract accumulating changes and damages. Senescence may, therefore, begin for protective reasons, but over time, it limits tissue and cells' regenerative capabilities. Since MSCs are increasingly recognized as the “guardians” of tissue homeostasis for their plastic response to injury, it is of great value to dissect the mechanisms that govern MSCs health. Moreover, MSCs can be exploited in regenerative medicine approaches after culture expansion. Reviewing the hallmarks of MSC changes associated with senescence highlights a central role of the redox control. The latter is associated with epigenetic changes that can provide a useful tool for selecting regenerative cells.

## Abbreviations Used

ARE: antioxidant responsive elements
CAD: coronary artery disease
EC: endothelial cell
eNOS: endothelial nitric oxide synthase
ESS: Epigenetic-Senescence-Signature
GMP: good manufacturing practices
lncRNA: long noncoding RNA
LVEF: left ventricular ejection fraction
MHC: major histocompatibility class
MCP-1: monocyte chemoattractant potein-1
miR: microRNA
MPs: muscular pericytes
PKC: protein kinase C
RBP: RNA-binding protein
ROS: reactive oxygen species
SASP: senescence-associated secretory phenotype
SC: stem cell
SIRT1: sirtuin 1
SOD: superoxide dismutase
TERT: telomerase reverse transcriptase
VSMCs: vascular smooth muscle cells
YMSC: young mesenchymal cell
